# Plant Glutathione Peroxidases: Non-Heme Peroxidases with Large Functional Flexibility as a Core Component of ROS-Processing Mechanisms and Signalling

**DOI:** 10.3390/antiox11081624

**Published:** 2022-08-21

**Authors:** Krisztina Bela, Riyazuddin Riyazuddin, Jolán Csiszár

**Affiliations:** 1 Department of Plant Biology, Faculty of Science and Informatics, University of Szeged, Közép fasor 52., H-6726 Szeged, Hungary; 2 Institute of Plant Biology, Biological Research Centre, Temesvári krt. 62., H-6726 Szeged, Hungary

**Keywords:** antioxidants, glutathione peroxidases, growth, reactive oxygen species, redox status, stress responses, thiol peroxidases

## Abstract

Glutathione peroxidases (GPXs) are non-heme peroxidases catalyzing the reduction of H_2_O_2_ or organic hydroperoxides to water or corresponding alcohols using glutathione (GSH) or thioredoxin (TRX) as a reducing agent. In contrast to animal GPXs, the plant enzymes are non-seleno monomeric proteins that generally utilize TRX more effectively than GSH but can be a putative link between the two main redox systems. Because of the substantial differences compared to non-plant GPXs, use of the GPX-like (GPXL) name was suggested for *Arabidopsis* enzymes. GPX(L)s not only can protect cells from stress-induced oxidative damages but are crucial components of plant development and growth. Due to fine-tuning the H_2_O_2_ metabolism and redox homeostasis, they are involved in the whole life cycle even under normal growth conditions. Significantly new mechanisms were discovered related to their transcriptional, post-transcriptional and post-translational modifications by describing gene regulatory networks, interacting microRNA families, or identifying Lys decrotonylation in enzyme activation. Their involvement in epigenetic mechanisms was evidenced. Detailed genetic, evolutionary, and bio-chemical characterization, and comparison of the main functions of GPXs, demonstrated their species-specific roles. The multisided involvement of GPX(L)s in the regulation of the entire plant life ensure that their significance will be more widely recognized and applied in the future.

## 1. Introduction

The generation of reactive oxygen species (ROS), such as superoxide radical (O_2_^•−^), hydrogen peroxide (H_2_O_2_), singlet oxygen (^1^O_2_), and hydroxyl radical (OH^•^), is a by-product of aerobic life. These highly reactive compounds are constantly produced, essentially by respiratory and photosynthetic electron transport chains, and can react with biomolecules including lipids, proteins, and nucleic acids [1,2]. ROS and reactive nitrogen species (RNS) may trigger several post-translational modifications, such as disulfide bond formation, thiol oxidation to sulfenic/sulfinic/sulfonic acid, glutathionylation or nitrosylation. Since an elevated ROS level can trigger damage or irreversible effects on development of tissues and organs, different non-enzymatic antioxidants (such as ascorbate, glutathione, carotenoids, tocopherols) and ROS-processing enzymes have evolved in aerobic organisms [2].

**Figure 1:**
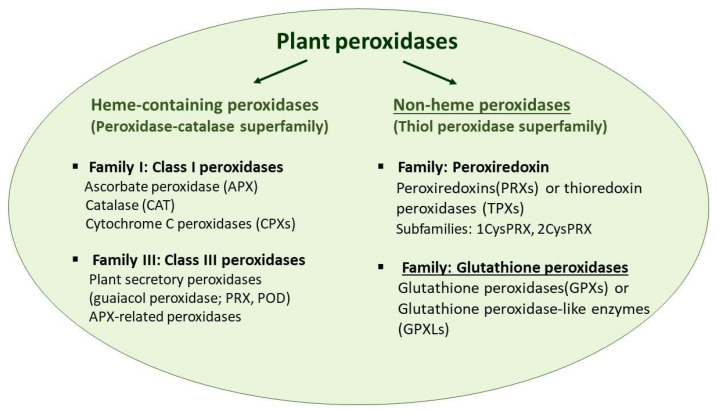
Schematic representation of classification of plant peroxidases using information from the RedoxiBase database (https://peroxibase.toulouse.inra.fr, accessed on 22 June 2022) and in [3,10].

The extremely widespread and diversified H_2_O_2_ decomposing peroxidases (EC.1.11.1.x) are present in all living organisms (reviewed, e.g., in [3]). They can be grouped based on the heme cofactor [4,5]. According to the RedoxiBase database, more than 80% of known peroxidase genes code heme peroxidases (https://peroxibase.toulouse.inra.fr, accessed on 22 June 2022). In plants, the most widely known peroxidases—such as the ascorbate peroxidase and catalase belonging to the intracellular Class I peroxidases and guaiacol peroxidases, the Class III peroxidases secreted to the extracellular space or transported into the vacuole—are heme-containing enzymes that are in the peroxidase-catalase superfamily [3,4,6]. The importance of non-heme peroxidases has emerged in the last decades [7,8,9]. The non-heme peroxidases comprise thiol peroxidases, alkylhydroperoxidase, haloperoxidases, NADH peroxidases and the pseudocatalase manganese catalases; but only the members of thiol peroxidase superfamily have been described in plants (Figure 1) [4,6] (https://peroxibase.toulouse.inra.fr, accessed on 22 June 2022). Among them, the ubiquitous thiol peroxidases serve both as ROS scavengers and contributors of ROS signalling. They are divided into two main enzyme families: peroxiredoxins (PRXs) or thioredoxin peroxidases, and glutathione peroxidases (mostly abbreviated as GPXs or GPxs).

GPXs (EC 1.11.1.9 for classical glutathione peroxidase and EC 1.11.1.12, phospholipid-hydroperoxide glutathione peroxidase) differ substantially both for the oxidizing peroxides and the reducing substrates [11]. They catalyze the reduction of H_2_O_2_ or organic hydroperoxides to water or corresponding alcohols and oxidize reduced glutathione (GSH, γ-Glu-Cys-Gly) or thioredoxin (TRX) [12,13]. The first GPX was discovered in erythrocytes [14], but later several GPXs were described in all estimated eukaryotic organisms. Some of the GPX isoenzymes contain the highly reactive selenocysteine (SeCys) residue in their active site, while others contain Cys [15]. Both the seleno- or nonseleno GPXs are considered to be central components of ROS-processing mechanisms in animals [13,16]. Mammals harbour eight GPX isoenzymes (GPX1-8), of which five (GPX1-4 and GPX6 in human) contain SeCys in their active site, and three (GPX5, GPX7, and GPX8) employ active-site cysteines [17,18,19]. They are crucial players in many biological processes, such as fertility, anti-inflammatory and anti-carcinogenesis associated routes [18,20,21]. It was suggested that the convergent expansion of mammalian GPXs in independent lineages might be important for avoiding oxidative damages and the adaption to stressful environments [22]. GPX4, otherwise called phospholipid hydroperoxide glutathione peroxidase (PHGPX) and originally peroxidation inhibiting protein (PIP), participates especially in the maintenance of membrane integrity due to decreasing the amount of lipid peroxides, and it has key role in the regulation of ferroptosis [23,24,25].

The plant GPXs exhibit the highest homology to the animal GPX4 isoenzyme; however, the plant enzymes contain Cys instead of SeCys in their active site and generally prefer the TRX regenerating system rather than GSH [8,17,26,27]. Due to their structural similarity to animal GPXs, but different activities and substrate specificities, the glutathione peroxidase-like (GPXL) name was suggested for the *Arabidopsis thaliana* GPX isoenzymes [10]. Besides keeping low ROS level, the ROS-processing antioxidant enzymes may even sense and signal ROS availability and redox perturbations [28]. They are involved in control of ROS gradients e.g., in the maintenance of stem cell niche or triggering differentiation in the shoot and root apical meristems (SAM and RAM, respectively), and in the proper zygote/embryo development [29,30,31,32]. In addition, using GSH and/or TRX as a reductant, the GPX(L)s influence the redox status of these main redox compounds. They can modify the thiol/disulfide balance and protein activity and moreover were considered to function as redox sensors by linking ROS to functional redox signalling [27,33,34,35,36].

## 2. Phylogenetic Aspects of Plant GPXs

Since the GPXs present no linear evolution, and non-animal GPXs are very distinct from most vertebrate GPXs, the original ancestor of the GPX gene family is uncertain [15,17]. Based on through robust phylogenetic studies and sequence analyses, Trenz et al. proposed that all GPX-encoding genes share a monomeric common ancestor and the bacterial, animal and the TRX-applying fungal and plant GPXs diverged early in evolution and diversified independently in different kingdoms and phyla [15]. This might explain the findings that, e.g., the *Tetrahymena thermophila*, a unicellular eukaryote (a ciliate) genome contains 12 *GPX* genes [37], but the *Chlamydomonas reinhardtii* unicellular green alga employs two SeCys-containing GPXs and three non-selenium GPXs (GPX3-5) [9,38]. Phylogenetic studies of *GPX* genes from different plant species showed that their number varies between 2 and 25 [39,40,41]. For example, two *GPX* genes were identified in *Physcomitrella patens* [39] and *Panax ginseng* [42], three in *Hordeum vulgare* [43] and *Vigna radiata* [44], four in *Pinus tabulaeformis* [45] and *Brachypodium distachyon* [39], and five genes in *Oryza sativa* [16,39], *Phoenix dactylifera* (date palm) [46], *Populus trichocarpa* [26], *Ricinus communis* [47], and *Solanum lycopersicum* [39]. Six GPXs are encoded by *Cucumis sativus* [48], *Citrullus lanatus* [49] and *Lotus japonica* [50] genome, seven genes were found in *Sorghum bicolor* [51] and *Zea mays* [52], while there are eight in *Arabidopsis thaliana* [53] and *Brassica oleracea* [40]. It was concluded that *GPX* genes showed duplication events in many plant species, e.g., in *Arabidopsis* [53] and maize [52]. In most of the cases, a relatively higher number of *GPX* genes was found in plants with polyploid genome [54]. For example, 12 genes were identified in *Brassica rapa* [40] and *Triticum aestivum* [55], 13 in *Gossypium hirsutum* [54] and *Glycine max* [44], and 25 *GPX* genes in *Brassica napus* [40].

According to the conserved domain and gene structure analyses conducted on *GPX(L)* genes from various species, the plant *GPXs* can be categorized into four or five main groups [44,47,52,54,55,56,57,58]. Comparison of *GPX* genes belonging to distinct groups disclosed highly similar motifs and conserved exon-intron arrangement patterns within each group [52]. This indicates that their structure and function might have been preserved during evolution, yet several differences were also discovered, like in *R. communis* and *Z. maize* [47,52]. Generally, the number of exons ranges between four to six, and introns numbers varied from four to ten but showed significant variability among species (e.g., [44,52,54,55,58]. Deviations might be assigned to gene and whole-genome duplications. Evidence of tandem or segmental duplications has been found at several plant *GPXs* [39,40,44,52,53]. It was suggested that the gene replication activities might play a crucial role in gene evolution [40].

## 3. Structure, Biochemical Properties, and Main Activities of Plant GPX Proteins

Plant GPXs are monomeric proteins (Table 1). The conserved protein structure of GPXs consists of central β-sheets surrounded by α-helices [59]. Most of the mammalian GPXs possess an oligomerization loop between the α3 helix and β6 strand, and consequently they form dimers or tetramers [60], however the monomer mammalian GPX4 (PHGPX) and plant GPXs do not contain any oligomerization loop. Although it was reported that *P. trichocarpa* GPX5 can also form a dimer, in this case the dimerization occurs due to non-covalent bonds with the help of hydrophobic and aromatic residues [59].

Sub-cellular localization analyses in various species revealed that the GPXs are localized in chloroplasts, mitochondria, cytoplasmic, extracellular and nuclear regions [16,51,52,54,55]. Although it was proposed that in other cellular compartments, such as peroxisomes and endoplasmic reticulum (ER), other antioxidant enzymes are the main ROS scavengers [52], Attacha et al. proved that AtGPXL3 is a luminal protein that can be anchored to the ER and Golgi membranes [10]. The presence of a transmembrane domain was reported too for example in corn ZmGPX4 enzyme [52].

The catalytic mechanism of glutathione peroxidases is the following:

2 GSH + H_2_O_2_ → glutathione disulfide + 2 H_2_O

2 GSH + lipid hydroperoxide → glutathione disulfide + lipid + 2 H_2_O

It was suggested that the monomer structure allows the direct reduction of membrane-bound lipid peroxides [20,63], thus the main proposed role of plant GPXs was in the maintenance of membrane integrity, especially under different stress conditions. Recent results of the molecular docking studies performed on maize proteins with three lipid hydroperoxides also strengthen this function [52]. Interestingly, the reduction activity of purified *Arabidopsis*, sunflower, and tomato GPXs with H_2_O_2_ were similar or even 2–7-times higher than those with organic hydroperoxides using *Escherichia coli* TRX [27,61]. In contrary to the yeast GPX, among the investigated recombinant plant GPXs (AtGPXL1, −2, −5, −6, HaGPX1, SlGPX1 and one *B. rapa* GPX) none of them utilized GSH for reduction of H_2_O_2_. These enzymes showed generally higher preference towards lipid hydroperoxides as electron acceptors and, except for *A. thaliana* and *B. rapa* GPX(L)s, they accepted GSH as electron donor (Table 1), but showed very low activity [27,61]. These results, together with the similar function of GPXs in model and crop plants (detailed later in Section 4 and Section 5) may justify the use of the GPXL name and abbreviation for all plant GPX proteins [10,64]. Interestingly, the levels of the lipid peroxidation marker malondialdehyde (MDA) and/or H_2_O_2_ were increased in several *Arabidopsis gpxl* mutants [65,66,67], indicating that these enzymes in vivo participate both in conversion of lipid hydroperoxides to less toxic molecules and are involved in the H_2_O_2_ homeostasis.

During the reduction of peroxides, the catalytic CysP-S- is oxidized to a sulfenic acid (CysP-SOH). The main difference between the distinct classes of non-heme peroxidases is the mechanism of regeneration of the CysP-SOH, which can be reduced directly (1-Cys mechanism) or by involving a second, so-called resolving Cys residue (CysR-SH) of the enzyme (2-Cys catalytic cycle) [7]. Trenz et al. suggested that the ancestral GPX protein contained both the peroxidatic and resolving cysteines [15]. In plant GPXs, the sulfenic acid forms an intramolecular disulfide with a second Cys. However, beside the two catalytic cysteines, the plant GPXs contain a third conserved Cys residue outside of the classical catalytic site, but its function is still not clear. In some cases, both the second and the third Cys can be responsible for disulfide bridge formation, as it was reported in Chinese cabbage [62], while in poplar the third Cys is the resolving type [26]; nevertheless, these are not general features of plant GPXs. 

The 2-Cys disulfide can be reduced by GSH or by TRX [68]. Kinetic characterization of recombinant proteins originating from diverse sources revealed that the activity (depending on the used peroxide substrates and plants) was much higher in the presence of TRX than that of GSH [26,27,61,62]. The investigated *Arabidopsis* enzymes were able to reduce the peroxide only with TRX [27]. The intramolecular rearrangement, catalytic cycle and regeneration of plant GPXs are similar to that of the peroxiredoxins, thus they were even suggested to be considered as the fifth group of PRXs [7,26]. 

In vivo activity measurements conducted on different *Arabidopsis* T-DNA insertion mutants revealed that the single mutation of *AtGPXL* genes could significantly decrease the TRX activity especially in shoots both under control conditions and after applying salt stress [64]. Interestingly, in the *AtGPXL5* overexpressing plants (OX-AtGPXL5), the glutathione peroxidase and thioredoxin peroxidase activities (GPOX and TPOX, respectively) were not elevated compared to the wild type under the above conditions [67,69]. It should be noted that the most numerous, plant-specific classes of the diverse glutathione transferase (GST) enzyme family exhibit more GSH-dependent peroxidase activities than GPXs against H_2_O_2_ and organic peroxides [70]. In addition, GPXs possess some functional overlaps with the PRXs, thus GPXs were suggested to be a putative link between the glutathione- and the thioredoxin-based detoxifying systems [53,56,62].

However, the involvement of GPXs is indicated not only in ROS detoxification but also in protection of cellular redox homeostasis by regulation of the thiol/disulfide balance and protein functions [27]. Meyer et al. [56] proposed that thiol peroxidases link ROS to functional redox signalling [36]. GPXs can oxidase Cys-containing proteins involved in the signalling, such as phosphatases, kinases, and transcription factors, thus regulating different pathways [27,56,71,72]. Even more, the significance of ER-localized GPXL3 in oxidative protein folding, in disulfide bridge formation and/or regeneration of the participant enzymes, at the same time processing the H_2_O_2_ arose locally, were implicated [10,36]. As a summation, plant GPXs might have innumerable roles in stress tolerance and development [41].

## 4. Involvement of GPXs in the Signalling Crosstalk under Abiotic Stress Responses

Investigation of the spatiotemporal expression levels of plant *GPX* genes revealed that they are mainly induced, but some of them are downregulated in response to various stresses [16,33,52,56,64]. The literature evidence hints that alteration of *GPXs* gene expression levels under different environmental stresses, such as salt stress, drought stress, temperature stress (high and cold), metals stress, as well as under biotic stress, was reported in several plant species [26,53,58,73]. The possible contribution of GPX isoenzymes in abiotic and biotic stress tolerance of plants was also indicated mostly by upregulation of enzymatic and non-enzymatic antioxidant defense mechanisms [34,64,67,74].

Glutathione peroxidase enzymes might be involved in the signalling crosstalk during abiotic stress responses via redox signal transduction, epigenetic regulation, transcription factors and direct protein–protein interactions. Based on the literature, GPXs can interact with other proteins and therefore they are considered to have signalling functions [33,75]. For example, AtGPXL3 interacts with 2C type protein phosphatase abscisic acid insensitive 1 and 2 (ABI1 and 2), therefore it acts an oxidative signalling transducer in ABA and drought stress signalling by stimulating the stomata closure via the activation of plasma membrane Ca^2+^ and K^+^ channels [33]. Recently, Paiva and co-workers confirmed the role of OsGPX3 in antioxidants defense, regulation of redox homeostasis and ABA signalling pathway in the rice plants [76]. Alternatively, AtGPXL3 also interacts with other transcription factors such as dehydration-responsive element-binding protein (DREB2A and DREB2B) via CEO1 interacting protein that controls the genes involved in plant responses to dehydration and heat stress [33]; ultimately, GPXL3 could act as a redox modulator of other proteins, influencing various critical metabolic processes. In *O. sativa*, the involvement of mitochondrial GPX1 and GPX3 in signalling between respiration and photosynthesis processes under normal and salt stress conditions were described [74,77]. Silencing of *OsGPX1* triggered impairment of photosynthesis, elevated H_2_O_2_ and decreased GSH contents, and in parallel reduced shoot growth and seed numbers were detected compared to wild type plants [77]. The *OsGPX3*-silenced plants showed decreased chlorophyll content, photosystem II activity, CO_2_ assimilation rate, stomatal conductance, intercellular CO_2_ partial pressure and higher H_2_O_2_ content in roots [78]. It was suggested that mitochondrial GPX deficiency resulted in redox changes, and OsGPX1 and −3 can act as a molecular regulator of crosstalk between chloroplasts and mitochondria due to altering the redox status [78]. Some reports in *Arabidopsis* have evidenced that chloroplastic GPXL isoforms are important to regulation of redox homeostasis and protection against oxidative stress generated by salinity [53]. It has been reported that GPXL7 maintained the photosystem II in *A. thaliana* plants via interacting with putative high chlorophyll fluorescence protein (HCF244), which participated in the biogenesis of PSII under high light-induced photooxidative stress [79]. In *gpxl7* mutant, the accumulation of HCF244 and D1 proteins were downregulated, and furthermore the plant became hypersensitive to H_2_O_2_ treatment [79]. Gaber et al. [67] described that the nucleus-localized AtGPXL8 isoenzyme not only protects the cellular compartments against oxidative damage, but was also involved in redox modification of proteins, therefore taking part in nucleus signal transduction [80].

Beside their role in stress tolerance and ABA signalling, GPXs also regulate the epigenetic processes. For instance, OsGPX3 might have a possible role in epigenetic regulation due to DNA methylation [78]. In *A. thaliana*, methylation of *GPXL1* histone by PRMT4b subsequently enhanced the expression of *GPXL1*, and the encoded antioxidant enzyme helped in the alleviation of paraquat-induced oxidative stress [81]. Interestingly, in rice plants, a proteomic approach revealed that silencing of *OsGPX3* negatively regulates the histone synthesis level, histone acetylase enzyme, and main enzymes responsible for further DNA processing, such as methylation, demethylation, assembly and remodelling of chromatin via induction of the S-glutathionylation of a putative protein (Uniprot code Q6Z8S7). This protein acts as a signal transducer and thus regulates the histone modification in the *Oryza sativa* plants [76]. Furthermore, Yang et al. [69] reported a new type of post-translational modification known as lysine decrotonylation, which occurred at the positions of Lys 220 of GPX1, increasing the glutathione peroxidase activity and thus minimizing the oxidative damage via reducing the level of cold-induced ROS, hence alleviating the cold stress in *Chrysanthemum morifolium* [82]. Mallikarjuna et al. [52] showed that four ZmGPX proteins (ZmGPX1, -3, -6, -7) have splice variants. Their differential expression in stress tolerant and sensitive genotypes under drought and waterlogging stresses indicates that the splicing mechanism targeted *ZmGPX* RNAs participate in the efficient stress responses [52,83].

Two putative *GPX* genes from *T. aestivum* were overexpressed in *A. thaliana* which led to altered transcript levels of genes involved in salt stress responses (*SOS1* and *RbohD*) and ABA-related regulation (*ABI1, ABI2*), thus implying the role of GPXs in salt and ABA signalling [84]. Our earlier results showed the alteration of the expression of transcription factors such as *DREB2A, DREB2B, MYC2* and that of 9-*cis*-epoxycarotenoid dioxygenase3 (*NCED3*) gene in *Atgpxl1-8* mutants both under normal conditions and after applying salt and osmotic stresses [64]. Alteration of several *AtGPXL* genes and selected stress-related transcription factor genes in the investigated *Atgpxl* mutants indicated their possible role in signalling to provide salt and osmotic stress tolerance [64]. The presence of *cis*-acting elements related to various abiotic stresses, biotic stress, and hormones in the 5′ up-regulatory regions of the *GPX(L)s* were reported [40,46,47,53,56,85].

In corn, 63 types of *cis*-acting elements were identified in the promoter regions of the seven *ZmGPX* genes, and among the regulating transcription factors were found C2H2, DOF, GRAS, MIKC, MADS, TCP, TALE and WRKY transcription factors [52]. Aside from this, except for *ZmGPX2* and *ZmGPX5*, the corn *GPXs* are targeted by regulatory miRNAs. Seven miRNA families, i.e., miR166, miR169, miR172, miR395, miR529, miR1432, miR2275, were shown to interact with *ZmGPXs* [52]. Induction of some of these miRNA genes was related to H_2_O_2_ treatment or redox signalling (miR169 and miR395, respectively). It was indicated that downregulation of *ZmGPX* genes may result in elevated H_2_O_2_ production [52]. Earlier, Li et al. [26] discovered five miRNAs from miR164 and miR396 families targeting six *BnGPX* genes [40].

Comparison of the 5′ regulatory region of each *Arabidopsis* and *T. salsuginea GPXLs* showed that they contain many *cis*-regulatory elements that were responsive to methyl jasmonate (MeJA), gibberellin (GA), auxin, ethylene (ET), salicylic acid (SA), drought, low temperature, and other abiotic stresses. Moreover, a greater number of *cis*-acting regulatory elements related to stress and hormone response were found in the promoter region of the salt-tolerant *Thellungiella GPXLs* compared to *AtGPXLs* [58]. Thus, GPXLs can be involved via *cis*-acting regulatory elements related to stress and hormone signalling to confer high stress tolerance to plants. In-silico-based prediction revealed that in the promoter region of *Ammopiptanthus nanus GPXs*, 40 *cis*-acting elements occur mostly participating in (a)biotic stress tolerance and hormone signalling [86]. Furthermore, Li et al. [40] observed *cis*-elements in the promoter region of *B. napus GPXs*; among them, four, five and several other *cis*-elements were linked with stress-responsive elements (drought, low-temperature, light, and anaerobic induction), hormone-related elements (auxin, ABA, GA, MeJ, SA) and light-related elements, respectively [40]. Similarly, conserved *cis*-acting elements associated with (a)biotic stresses and hormone response were reported in the promoter region of *Theobroma cacao, Phoenix dactylifera*, *R. communis*, *G. hirsutum* and *Z. mays GPXs* [46,47,52,54,85].

**Figure 2:**
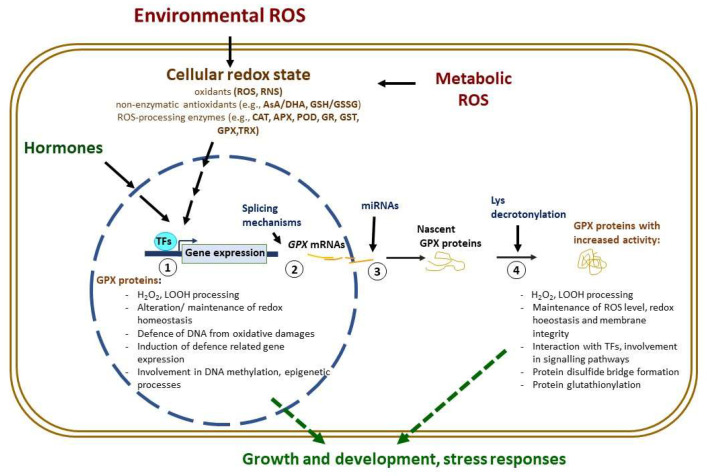
Schematic summary of regulation and main roles of cytoplasmic and nuclear localized plant glutathione peroxidases. (1) Transcriptional control of *GPX* gene expression via *cis*-regulatory elements and transcriptional factors, (2) post-transcriptional regulation of *GPX* mRNAs by splicing mechanisms, (3) *GPX* mRNAs can be targeted by different types of miRNAs, (4) Lys decrotonylation can increase the GPX protein activity. Abbreviations: APX, ascorbate peroxidase; AsA, ascorbic acid; CAT, catalase; DHA, dehydroascorbate; GPX, glutathione peroxidase; GR, glutathione reductase; GSH, reduced glutathione; GSSG, oxidized glutathione; GST, glutathione transferase; LOOH, lipid peroxide/hydroperoxide; mRNA, messenger RNA; miRNA, microRNA; POD, guaiacol peroxidase; RNS, reactive nitrogen species; ROS, reactive oxygen species; TFs, transcription factors; TRX, thioredoxin.

Hence, GPXs are involved in signalling during environmental stresses, and activity of GPXs is regulated at both the transcriptional and post-translational level (Figure 2).

## 5. GPXs Regulates the Growth and Development of Plants

**Figure 3:**
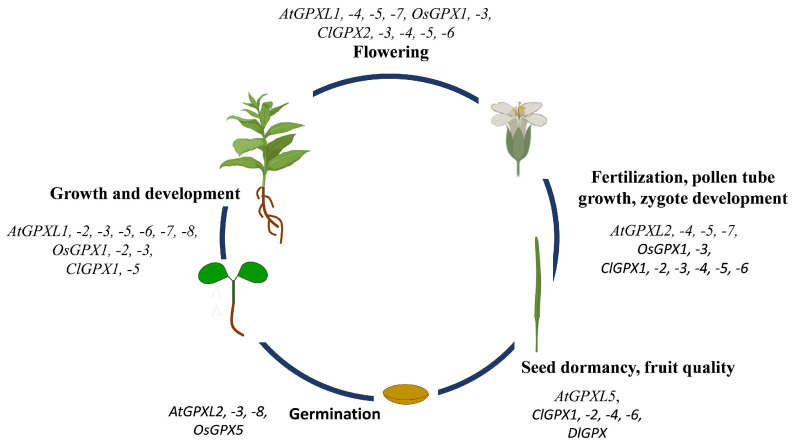
Schematic representation of involvement of plant GPX(L)s in growth and development in different model and crop plants based on the results published in [32,34,41,56,67,69,87,88]. *At*: *A. thaliana*, *Cl*: *C. lanatus*, *Dl*: *Dimocarpus longan*, *Os*: *O. sativa.* Some parts of the figure were created with BioRender.com (accessed on 7 August 2022).

Besides their role in stress tolerance, GPXs regulate plant growth and development under normal as well as in unfavorable conditions. The relevance of GPX(L)s in growth and development came to light after reports of the high transcript amount of *GPX(L)* genes in *O. sativa* and *A. thaliana* plants and that their expressions are dependent on tissues and developmental stages (Figure 3) [16,34,56,67,74]. Elevated expression levels of the *AtGPXL2, AtGPXL3,* and *AtGPXL8* genes were reported during the process of *Arabidopsis* seed germination, while the rest of them, such as *AtGPXL1, AtGPXL4, AtGPXL5, AtGPXL6,* and *AtGPXL7*, were downregulated [56]. Passaia et al. [74] reported that the knockdown of *OsGPX1* or *OsGPX3* severely affected the growth and development of rice plants [74]; furthermore, according to recent findings, they have crucial roles in development of zygotes and embryos [32].

Rattanawong et al. demonstrated that both GSH depletion and inhibition of GPX activity resulted in high ROS accumulation in zygotic/embryonic nuclei, impairing the proper early embryonic development. Their results indicate the cooperative roles of GSH and OsGPX1 in quenching of nuclear ROS to promote developmental progression of the zygote [32]. The regulator function of ROS was reported both in somatic tissues and reproductive processes, such as megagametogenesis, programmed cell death in tapetum, pollen–pistil interaction, pollen tube growth and early embryogenesis [29,31,32]. In addition, GSH regulates the division of the cell cycle, cell differentiation, and transition from G1 to S phase, while the conversely higher amount of GSSG leads to hampering of the cell proliferation further [89,90,91]. In the zygote, GSH can participate in decreasing the H_2_O_2_ level directly as a co-substrate for OsGPX1 or as part of the “Foyer-Halliwell-Asada” pathway [32]. Due to these ROS-related events, DNA integrity is achieved in the zygote, thereby progressing to the next phase of the cell cycle and subsequent cell division. Interestingly, the GPX1 activity is responsible for temporary accumulation of GSSG, which is also essential in the early embryogenesis. Earlier it was reported that plant GPXs preferentially utilize TRX as electron donor instead of GSH [27], and it has been demonstrated that GSH exhibits compensatory activity when the TRX reduction system (NADPH-dependent TRX reductase A and B genes) is impaired [92]. In contrary, convincing results of Rattanawong and co-workers’ experiments indicate the use of GSH in the GPX-catalyzed H_2_O_2_ reduction reaction in vivo in *O. sativa* zygotes [32]. Pagnussat et al. reported on similar functions of AtGPXLs: insertional *Atgpxl5* knock-out mutant led to the abruption of endosperm formation and considerable embryo lethality [93].

It is well established that cellular redox homeostasis, mainly depending on ROS, GSH/GSSG and AsA/DHA redox couples and related enzymatic antioxidants, is one of the key regulators of growth, development, organogenesis, and regeneration of cells in plants [29,89,94,95,96]. As another example, the development of root architecture is also determined by the differing redox status and the distribution of ROS in the meristematic and other root regions [31,97]. GSH participates in the activation and maintenance of cell division, especially in root apical cells [98,99,100,101]. The gene expression pattern analysis in *Arabidopsis* GSH-deficient *root meristemless 1-1* (*rml1-1*) mutant revealed altered expression levels of redox-related genes, such as *GSTs*, glutaredoxins (*GRXs*), h-type thioredoxins (*TRXhs*), and *GPXLs* [101]. During the acute shortage of GSH in *Arabidopsis* roots, a higher degree of TRX utilization compared to GSH was suggested, a hint toward the relationship between GSH and TRX systems [101]. The *rml1-1* roots also possessed lower transcript amount of *PIN5* auxin transporter and higher transcript level of *IAA20* that caused root meristem collapse [102,103]. On the contrary, lower expression of *RADIALIS-LIKE SANT/MYB 1(RMS1*) and *HOOKLESS 1 (HLS1*) genes were reported under GSH depletion conditions that are responsible for the control of early photomorphogenesis in *A. thaliana* plants [104]. Intriguingly, the shoot of *rml1-1* mutant plants was not significantly affected. This might be due to the thioredoxin-dependent control, since GSH and TRX systems are interconnected, as has been previously suggested [90,101,105]. Maintaining the reduced thiols homeostasis in plants by the TRX system is important for the regulation of root architecture, but also for chloroplast biogenesis, and development of leaves [106,107]. For example, a mutation in TRXs can lead to hampering the development of chloroplast, root, and leaves in *Arabidopsis* and tobacco plants [106,107,108,109].

Passaia and co-workers investigated the role of GPXLs in response to auxin, ABA, and strigolactone (SL) hormones by using T-DNA insertion mutants (*Atgpxl1-8*) and found the importance of these isoenzymes in the regulation of lateral root development through redox- and hormone-mediated pathways [34]. The role of GPXL7 in the hormone-dependent development of roots was proven by applying 1-naphtaleneacetic acid (NAA) and synthetic SL [34]. Furthermore, *gpxl7* knock-out mutants showed a significantly higher number of rosette leaves reported in short-day and long-day photoperiods, respectively, verifying the importance of AtGPXL7 in shoot development [34]. According to our recent results, lack of AtGPXL5 enzyme activity negatively influenced the plant growth and development by decreasing the length of primary roots, the biomass, the chlorophyll and anthocyanin pigment contents, rosette size, and convex area of leaves as compared to wild types and overexpressing lines under normal environmental conditions [67]. Additionally, the importance of GPXL5 in the development and skotomorphogenesis process of dark-grown *Arabidopsis* seedlings was demonstrated, as knock-down *Atgpxl5* mutants showed defective phenotypes, such as decreased growth of hypocotyl and radical compared to 4-day old dark-grown wild type and *AtGPXL5* overexpressing plants [69]. Although the elevated ROS level and more oxidized redox status of the *Atgpxl5* mutants can trigger the increase of the ET production, changes in the ET-related gene expression pattern both in the insertional mutant and the *AtGPXL5*-overexpressing plants indicate the crosstalk between AtGPXL5 and ethylene signalling [69].

The importance of GPX(L)s in the proper growth and development of model plants has enabled researchers to apply this knowledge to crop plants. For instance, it was found that a mutation in mitochondrial-localized OsGPX3 led to stunted growth of shoots and roots and negatively regulates the photosynthesis and seed production in rice plants as compared to wild types [74,77]. Another isoenzyme, OsGPX5 of rice plant, was studied by Wang et al. [110] and they reported that knock-out mutation of *OsGPX5* exhibited a lower germination rate, decreased growth, and less filling of grains and seed setting than the wild type plants (Figure 3) [110]. Recently, the tissue specific *GPX* gene expression level was reported in several crop plants (Table 2.) [40,52,55,111]. Among the 25 *BnGPXs* genes, group II genes such as *BnGPX1*−*14*, −*8*, −*18*, −*11*, −*25*, −*12*, and −*23* all were upregulated in shoot, roots, leaves, flower, silique, and seeds, except for the downregulation of *BnGPX8,* −*12*−*18* in the seeds [40]. However, genes belonging to other groups were downregulated instead of *BnGPX2,* −*4*, −*15*, −*22*, which were significantly higher in the leaves, flower, seeds, and silique. The high *GPX* expression levels indicate that these genes are very important in the developmental processes of rape seed [40]. During investigation of the redox regulation of *Dimocarpus longan* fruit senescence, Wu et al. also identified a GPX, which is involved in fruit senescence or quality deterioration of harvested *D. longan* fruit [88]. In *C. lanatus* the *ClGPX1,* −*3* and −*5* showed relatively high or moderate expression in expanding and mature leaves or roots, respectively [49]. Extremely high *ClGPX1* expression was measured in fruits, but the high transcript level of another five *ClGPX* genes in flowers and fruits indicated that the encoded proteins might play important roles in various physiological and developmental processes of watermelon [49].

The involvement of GPXs in the shoot organogenesis was also shown [117]. Introduction of transgenic lines with the overexpression of GPX from *Citrus sinensis* led to unsuccessful regeneration of plants, which might be due to uncontrolled hunting of ROS level by constitutive expressed GPX isoenzyme, as an optimum level of ROS is required for regeneration of shoots at the early stage of plants [117]. In short, GPXs are important regulators of the shoot and root development, but further clarification of their species-specific functions is needed. Although the heme-containing Class I and Class III peroxidases are much larger plant enzyme families, the non-heme GPX(L)s are also important ROS scavenging proteins; their species-specific functions may have more important signalling functions due to locally fine-tuning the ROS level and redox homeostasis or modifying the activity of interacting regulatory proteins (Table 3).

## 6. Conclusions

While animal GPXs are well-estimated enzymes, less information is available on plant GPXs. Although several enzymes have been purified and their biochemical properties have been analyzed, their in vivo roles and significance is still unexplored. Earlier it was thought that their main function is the conversion of lipid hydroperoxides into less toxic compounds and thus the maintenance of membrane integrity, in recent years their involvement in impacting the redox homeostasis and altering the H_2_O_2_ homeostasis and thiol/disulfide balance has come to the fore. In the last decades, much research has proved that plant GPX(L)s not only are essential elements of plant stress responses but are involved in several processes that determine the growth and development even under normal conditions. Thorough phylogenetic analysis of GPXs from different kingdoms has helped us to understand that the independent evolvement of genes led to their heterogeneous presence in genomes of plant relatives. Here we updated the main results of detailed molecular, biochemical, genetic, or phylogenetic analysis performed on this enzyme family from different plant sources that discovered several new interactions and functions (Figure 1). Among these, for example, their regulatory role in epigenetic processes is mostly unknown. In silico analysis of the promoter region of different plant *GPX* genes discovered the presence of different hormone- and light-responsive *cis*-regulatory elements beside the stress- and redox-associated sequences. Astonishing new findings were published related to the control of their post-transcriptional and post-translational regulation (via splicing mechanisms, miRNA driven silencing or Lys de-crotonylation, respectively) (Figure 1). Their general involvement in stress responses and the results obtained by overexpression of specific plant *GPX* genes foreshadow that these enzymes can be key players in the establishment of plants with increased stress tolerance. Overexpression of *GPX* genes in different plant species led to increased tolerance against different stresses, but also revealed their importance in the growth and developmental processes. *GPX* overexpression may be a promising approach in the molecular or traditional breeding to develop stress-resistant crop plants, however there are many unresolved questions. Firstly, it will be important to explore the species-specific roles and regulatory network of GPX(L)s in diverse crop plants under normal and stress conditions. Secondly, the crosstalk between GPX(L)s and other compounds of the antioxidant system and hormone signalling, though they influence stress responses, growth and developmental processes, still requires further intensive research. For example, the significance of GPX activation by de-crotonylation, or their involvement in the ferroptosis, are still unknown. Finally, it is also conceivable that due to their ability to catalyze redox reactions of different lipid hydroperoxides, plant GPXs might be used in the development of analytical and diagnostic kits, similarly to several members of the Class III peroxidase enzyme family, such as horseradish peroxidase (HRP). The multifaceted involvement in the regulation of physiological processes of the entire plant life ensures that the significant plant GPXs will be more widely recognized and applied in the future.

## Figures and Tables

**Table 1:** Biochemical properties of GPXs. AlkylOOH: alkyl hydroperoxide, CumOOH: cumene hydroperoxide, GSH: reduced glutathione, LOOH: lipid hydroperoxide, PCOOH: phoshatidylcholine hydroperoxide, PUFAOOH: polyunsaturated fatty acids hydroperoxide, ROOH: organic hydroperoxide, SeCys: Selenocysteine, TRX: thioredoxin.

	Protein Structure	Active Site Cys/SeCys	Reducing Agent	Substrate	References
**Animal GPXs**	tetramer, dimer, or monomer	SeCys or Cys	GSH, TRX, NADPH	H_2_O_2_, LOOH, ROOH	[11,12]
***A. thaliana* GPXLs**	monomer	Cys	TRX	AlkylOOH CumOOH PCOOH PUFAOOH	[27]
**GPXs from other plant species**	monomer (dimer)	Cys	TRX, GSH	AlkylOOH CumOOH PCOOH PUFAOOH	[26,32,52,61,62]

**Table 2:** Main reported functions of relevant GPXs from different mammalian and plant species.

Enzyme		Reported Function/Involvement				Organism	References
		Stress Responses	Redox Regulation/ Signalling	Normal Metabolism	Development		
**Animal GPXs**							
	HsGPX1, -2, -3, -5, -6	H_2_O_2_ and lipid hydroperoxide processing, stress tolerance	Insulin signalling	H_2_O_2_ and lipid hydroperoxide processing +	Male fertility	*Homo sapiens*	[112]
	HsGPX4	Lipid peroxidation, protein thiol oxidation	Cell death, Ferroptosis	Protein thiol oxidation	Spermatogenesis, chromatin condensation,	*Homo sapiens*	[23,24,25]
	HsGPX7	General scavenging of ROS, lipid peroxides Anti-inflammatory	Anti-carcinogenesis			*Homo sapiens*	[18,21,113]
	MmGPX4	Anti-carcinogenesis	Spermatogenesis		Spermatogenesis, male fertility, embryogenic development	*Mus musculus*	[20]
***A. thaliana*** **GPXLs**	AtGPXL1-8	Diverse biotic and abiotic (heat, cold, salt, drought, osmotic and metal) stresses, ferroptosis	Maintenance of redox homeostasis, oxidative signal transducer in ABA and drought stress signalling,	Photosynthesis	Development in whole life cycle (embryogenesis, germination, root, shoot apical meristem, hypocotyl, root system, rosette leaves, flowering, pollen tube growth, fertilization, seed dormancy)	*A. thaliana*	[33,36,41,53,58,64,67,69,114,115]
**GPXs from other plant species**	OsGPX1-5	Drought, salt, cold, oxidative stresses	Redox signalling, participation in the interaction between ER stress and redox homeostasis, crosstalk between mitochondria and chloroplast	Photosynthesis and cellular respiration	In development during the whole life cycle (embryogenesis, germination, root-, shoot apical meristem, hypocotyl, root system, seedling development, rosette leaves, inflorescence and silique, pollen tube growth, seed setting, grain filling, seed dormancy)	*O. sativa*	[16,32,34,39,74,77,87,110]
	SlGPX1-5 (GSHPxle1-5)	Heat stress, cold, light stress	unknown	unknown	unknown	*S. lycopersicum*	[39,58,116]
	HvGPX1-3	Oxidative stress, salt/osmotic stress, norflurazon, and paraquat resistance	unknown	unknown	unknown	*H. vulgare*	[43]
	BoGPX1-8	Salinity, cold, waterlogging, and drought	Bn *BoGPX* genes might contribute to stress responses and hormone signaling pathways	unknown	In development of root, seed, leaf, stem, flower, and silique	*B. oleracea*	[40]
	BrGPX1-12	Salinity, cold, waterlogging, and drought	*BrGPX* genes might contribute to stress responses and hormone signaling pathways	unknown	In development of root, seed, leaf, stem, flower, and silique	*B. rapa*	[40]
	BnGPX1-25	Salinity, cold, waterlogging, and drought	*BnGPX* genes might contribute to stress responses and hormone signaling pathways	unknown	In development of root, seed, leaf, stem, flower, and silique	*B. napus*	[40]
	GhGPX1-13	Salt stress, heat, sulphate solution	Importance of GhGPXs in hormone signalling, regulation of redox homeostasis	unknown	In regulation of plant growth and development	*G. hirsutum*	[54]
	TaGPX1-12	Heat, drought and/or a combination, salt	Possible role of *TaGPXs* in development and stress response, putative roles in signal transductions	GSH biosynthetic and metabolic processes, DNA metabolic processes	Putative roles in plant growth and development, in leaf developmental stages, roots, stems, spikes, and grain	*T. aestivum*	[55]
	ZmGPX1-7	Drought, waterlogging	Stress regulation through regulatory elements and splicing mechanisms		Growth, development	*Z. mays*	[52]

**Table 3:** Comparison of the involvement of heme-containing Class III peroxidases and the non-heme GPX(L) enzyme family in stress responses, growth and developmental processes and interactions with auxin and ethylene hormones.

Function/Involvement in:	Heme-Peroxidases Family: Class III Peroxidases	Non-Heme Peroxidases Family: Glutathione Peroxidases
ROS metabolism	+	+
Redox signalling	+	+
Defence against pathogen infection	+	+
Defence against abiotic stresses	+	+
Wound healing	+	−
Cell wall metabolism	+	−
Lignification and suberization	+	−
Defence of membranes	−	+
Growth and development	+	+
Seed germination	+	+
Growth of roots	+	+
Growth of shoots	+	+
Flowering	+	+
Fertilization, pollen tube growth	+	+
Embryogenesis, seed development	+	+
Fruit growth and ripening	+	+
Interaction with plant hormones	+	+
Auxin catabolism	+	−
Auxin transport	−	+
Ethylene biosynthesis	+	+
Ethylene signalling	−	+

## References

[1] Mittler R. (2002). Oxidative Stress, Antioxidants and Stress Tolerance. Trends Plant Sci..

[2] Foyer C.H., Noctor G. (2005). Redox Homeostasis and Antioxidant Signaling: A Metabolic Interface between Stress Perception and Physiological Responses. Plant Cell.

[3] Pandey V.P., Awasthi M., Singh S., Tiwari S., Dwivedi U.N. (2017). A Comprehensive Review on Function and Application of Plant Peroxidases. Biochem. Anal. Biochem..

[4] Passardi F., Cosio C., Penel C., Dunand C. (2005). Peroxidases Have More Functions than a Swiss Army Knife. Plant Cell Rep..

[5] Koua D., Cerutti L., Falquet L., Sigrist C.J.A., Theiler G., Hulo N., Dunand C. (2009). PeroxiBase: A Database with New Tools for Peroxidase Family Classification. Nucleic Acids Res..

[6] Passardi F., Theiler G., Zamocky M., Cosio C., Rouhier N., Teixera F., Margis-Pinheiro M., Ioannidis V., Penel C., Falquet L. (2007). PeroxiBase: The Peroxidase Database. Phytochemistry.

[7] Rouhier N., Jacquot J.-P. (2005). The Plant Multigenic Family of Thiol Peroxidases. Free Radic. Biol. Med..

[8] Herbette S., Roeckel-Drevet P., Drevet J.R. (2007). Seleno-independent Glutathione Peroxidases: More than Simple Antioxidant Scavengers. FEBS J..

[9] Dayer R., Fischer B.B., Eggen R.I.L., Lemaire S.D. (2008). The Peroxiredoxin and Glutathione Peroxidase Families in Chlamydomonas Reinhardtii. Genetics.

[10] Attacha S., Solbach D., Bela K., Moseler A., Wagner S., Schwarzländer M., Aller I., Müller S.J., Meyer A.J. (2017). Glutathione Peroxidase-like Enzymes Cover Five Distinct Cell Compartments and Membrane Surfaces in Arabidopsis Thaliana. Plant Cell Environ..

[11] Flohé L., Toppo S., Orian L. (2022). The Glutathione Peroxidase Family: Discoveries and Mechanism. Free Radic. Biol. Med..

[12] Arthur J.R. (2001). The Glutathione Peroxidases. Cell. Mol. Life Sci. C.

[13] Imai H., Nakagawa Y. (2003). Biological Significance of Phospholipid Hydroperoxide Glutathione Peroxidase (PHGPx, GPx4) in Mammalian Cells. Free Radic. Biol. Med..

[14] Mills G.C. (1957). Hemoglobin Catabolism: I. Glutathione Peroxidase, an Erythrocyte Enzyme Which Protects Hemoglobin from Oxidative Breakdown. J. Biol. Chem..

[15] Trenz T.S., Delaix C.L., Turchetto-Zolet A.C., Zamocky M., Lazzarotto F., Margis-Pinheiro M. (2021). Going Forward and Back: The Complex Evolutionary History of the GPx. Biology.

[16] Islam T., Manna M., Kaul T., Pandey S., Reddy C.S., Reddy M.K. (2015). Genome-Wide Dissection of *Arabidopsis* and Rice for the Identification and Expression Analysis of Glutathione Peroxidases Reveals Their Stress-Specific and Overlapping Response Patterns. Plant Mol. Biol. Rep..

[17] Margis R., Dunand C., Teixeira F.K., Margis-Pinheiro M. (2008). Glutathione Peroxidase Family—An Evolutionary Overview. FEBS J..

[18] Brigelius-Flohé R., Maiorino M. (2013). Glutathione Peroxidases. Biochim. Biophys. Acta BBA-Gen. Subj..

[19] Benhar M. (2018). Roles of Mammalian Glutathione Peroxidase and Thioredoxin Reductase Enzymes in the Cellular Response to Nitrosative Stress. Free Radic. Biol. Med..

[20] Conrad M., Moreno S.G., Sinowatz F., Ursini F., Kolle S., Roveri A., Brielmeier M., Wurst W., Maiorino M., Bornkamm G.W. (2005). The Nuclear Form of Phospholipid Hydroperoxide Glutathione Peroxidase Is a Protein Thiol Peroxidase Contributing to Sperm Chromatin Stability. Mol. Cell. Biol..

[21] Brigelius-Flohe R., Kipp A. (2009). Glutathione Peroxidases in Different Stages of Carcinogenesis. Biochim. Biophys. Acta BBA-Gen. Subj..

[22] Tian R., Geng Y., Yang Y., Seim I., Yang G. (2021). Oxidative Stress Drives Divergent Evolution of the Glutathione Peroxidase (GPX) Gene Family in Mammals. Integr. Zool..

[23] Krümmel B., Plötz T., Jörns A., Lenzen S., Mehmeti I. (2021). The Central Role of Glutathione Peroxidase 4 in the Regulation of Ferroptosis and Its Implications for Pro-Inflammatory Cytokine-Mediated Beta-Cell Death. Biochim. Biophys. Acta BBA-Mol. Basis Dis..

[24] Ursini F., Travain V.B., Cozza G., Miotto G., Roveri A., Toppo S., Maiorino M. (2022). A White Paper on Phospholipid Hydroperoxide Glutathione Peroxidase (GPx4) Forty Years Later. Free Radic. Biol. Med..

[25] Weaver K., Skouta R. (2022). The Selenoprotein Glutathione Peroxidase 4: From Molecular Mechanisms to Novel Therapeutic Opportunities. Biomedicines.

[26] Navrot N., Collin V., Gualberto J., Gelhaye E., Hirasawa M., Rey P., Knaff D.B., Issakidis E., Jacquot J.-P., Rouhier N. (2006). Plant Glutathione Peroxidases Are Functional Peroxiredoxins Distributed in Several Subcellular Compartments and Regulated during Biotic and Abiotic Stresses. Plant Physiol..

[27] Iqbal A., Yabuta Y., Takeda T., Nakano Y., Shigeoka S. (2006). Hydroperoxide Reduction by Thioredoxin-specific Glutathione Peroxidase Isoenzymes of *Arabidopsis thaliana*. FEBS J..

[28] Noctor G., Reichheld J.-P., Foyer C.H. (2018). ROS-Related Redox Regulation and Signaling in Plants. Proceedings of the Seminars in Cell & Developmental Biology.

[29] Foyer C.H., Wilson M.H., Wright M.H. (2018). Redox Regulation of Cell Proliferation: Bioinformatics and Redox Proteomics Approaches to Identify Redox-Sensitive Cell Cycle Regulators. Free Radic. Biol. Med..

[30] Huang H., Ullah F., Zhou D.X., Yi M., Zhao Y. (2019). Mechanisms of ROS Regulation of Plant Development and Stress Responses. Front. Plant Sci..

[31] Mase K., Tsukagoshi H. (2021). Reactive Oxygen Species Link Gene Regulatory Networks During *Arabidopsis* Root Development. Front. Plant Sci..

[32] Rattanawong K., Koiso N., Toda E., Kinoshita A., Tanaka M., Tsuji H., Okamoto T. (2021). Regulatory Functions of ROS Dynamics via Glutathione Metabolism and Glutathione Peroxidase Activity in Developing Rice Zygote. Plant J..

[33] Miao Y., Lv D., Wang P., Wang X.-C., Chen J., Miao C., Song C.-P. (2006). An *Arabidopsis* Glutathione Peroxidase Functions as both a Redox Transducer and a Scavenger in Abscisic Acid and Drought Stress Responses. Plant Cell.

[34] Passaia G., Queval G., Bai J., Margis-Pinheiro M., Foyer C.H. (2014). The Effects of Redox Controls Mediated by Glutathione Peroxidases on Root Architecture in *Arabidopsis thaliana*. J. Exp. Bot..

[35] Passaia G., Margis-Pinheiro M. (2015). Glutathione Peroxidases as Redox Sensor Proteins in Plant Cells. Plant Sci..

[36] Meyer A.J., Dreyer A., Ugalde J.M., Feitosa-Araujo E., Dietz K.-J., Schwarzländer M. (2021). Shifting Paradigms and Novel Players in Cys-Based Redox Regulation and ROS Signaling in Plants-and Where to Go Next. Biol. Chem..

[37] Ferro D., Bakiu R., Pucciarelli S., Miceli C., Vallesi A., Irato P., Santovito G. (2020). Molecular Characterization, Protein–Protein Interaction Network, and Evolution of Four Glutathione Peroxidases from Tetrahymena Thermophila. Antioxidants.

[38] Ma X., Zhang B., Miao R., Deng X., Duan Y., Cheng Y., Zhang W., Shi M., Huang K., Xia X.-Q. (2020). Transcriptomic and Physiological Responses to Oxidative Stress in a Chlamydomonas Reinhardtii Glutathione Peroxidase Mutant. Genes.

[39] Ozyigit I.I., Filiz E., Vatansever R., Kurtoglu K.Y., Koc I., Öztürk M.X., Anjum N.A. (2016). Identification and Comparative Analysis of H_2_O_2_-Scavenging Enzymes (Ascorbate Peroxidase and Glutathione Peroxidase) in Selected Plants Employing Bioinformatics Approaches. Front. Plant Sci..

[40] Li W., Huai X., Li P., Raza A., Mubarik M.S., Habib M., Fiaz S., Zhang B., Pan J., Khan R.S.A. (2021). Genome-Wide Characterization of Glutathione Peroxidase (GPX) Gene Family in Rapeseed (*Brassica napus* L.) Revealed Their Role in Multiple Abiotic Stress Response and Hormone Signaling. Antioxidants.

[41] Sharma A., Kaur A., Tyagi S., Upadhyay S.K. (2022). Glutathione Peroxidases in Plants: Innumerable Role in Abiotic Stress Tolerance and Plant Development. J. Plant Growth Regul..

[42] Kim Y.-J., Jang M.-G., Noh H.-Y., Lee H.-J., Sukweenadhi J., Kim J.-H., Kim S.-Y., Kwon W.-S., Yang D.-C. (2014). Molecular Characterization of Two Glutathione Peroxidase Genes of Panax Ginseng and Their Expression Analysis against Environmental Stresses. Gene.

[43] Churin Y., Schilling S., Börner T. (1999). A Gene Family Encoding Glutathione Peroxidase Homologues in *Hordeum*
*Vulgare* (Barley). FEBS Lett..

[44] Aleem M., Aleem S., Sharif I., Wu Z., Aleem M., Tahir A., Atif R.M., Cheema H.M.N., Shakeel A., Lei S. (2022). Characterization of SOD and GPX Gene Families in the Soybeans in Response to Drought and Salinity Stresses. Antioxidants.

[45] Zhao L., Han X.-M., Wang W., Yang H.-L. (2014). Molecular and Catalytic Properties of Glutathione Peroxidase Family Proteins from *Pinus*
*tabulaeformis*. Plant Mol. Biol. Rep..

[46] Jana G.A., Yaish M.W. (2020). Genome-Wide Identification and Functional Characterization of Glutathione Peroxidase Genes in Date Palm (*Phoenix*
*dactylifera* L.) under Stress Conditions. Plant Gene.

[47] Wang X., Liu X., An Y., Zhang H., Meng D., Jin Y., Huo H., Yu L., Zhang J. (2021). Identification of Glutathione Peroxidase Gene Family in Ricinus Communis and Functional Characterization of RcGPX4 in Cold Tolerance. Front. Plant Sci..

[48] Zhou Y., Hu L., Ye S., Jiang L., Liu S. (2018). Genome-Wide Identification of Glutathione Peroxidase (GPX) Gene Family and Their Response to Abiotic Stress in Cucumber. 3 Biotech.

[49] Zhou Y., Li J., Wang J., Yang W., Yang Y. (2018). Identification and Characterization of the Glutathione Peroxidase (GPX) Gene Family in Watermelon and Its Expression under Various Abiotic Stresses. Agronomy.

[50] Ramos J., Matamoros M.A., Naya L., James E.K., Rouhier N., Sato S., Tabata S., Becana M. (2009). The Glutathione Peroxidase Gene Family of Lotus Japonicus: Characterization of Genomic Clones, Expression Analyses and Immunolocalization in Legumes. New Phytol..

[51] Akbudak M.A., Filiz E., Vatansever R., Kontbay K. (2018). Genome-Wide Identification and Expression Profiling of Ascorbate Peroxidase (APX) and Glutathione Peroxidase (GPX) Genes under Drought Stress in Sorghum (*Sorghum bicolor* L.). J. Plant Growth Regul..

[52] Mallikarjuna M.G., Sharma R., Veeraya P., Tyagi A., Rao A.R., Chandappa L.H., Chinnusamy V. (2022). Evolutionary and Functional Characterisation of Glutathione Peroxidases Showed Splicing Mediated Stress Responses in Maize. Plant Physiol. Biochem..

[53] Milla M.A.R., Maurer A., Huete A.R., Gustafson J.P. (2003). Glutathione Peroxidase Genes in Arabidopsis Are Ubiquitous and Regulated by Abiotic Stresses through Diverse Signaling Pathways. Plant J..

[54] Chen M., Li K., Li H., Song C.-P., Miao Y. (2017). The Glutathione Peroxidase Gene Family in Gossypium Hirsutum: Genome-Wide Identification, Classification, Gene Expression and Functional Analysis. Sci. Rep..

[55] Tyagi S., Sembi J.K., Upadhyay S.K. (2018). Gene Architecture and Expression Analyses Provide Insights into the Role of Glutathione Peroxidases (GPXs) in Bread Wheat (*Triticum*
*aestivum* L.). J. Plant Physiol..

[56] Bela K., Horváth E., Gallé Á., Szabados L., Tari I., Csiszár J. (2015). Plant Glutathione Peroxidases: Emerging Role of the Antioxidant Enzymes in Plant Development and Stress Responses. J. Plant Physiol..

[57] Gupta S., Dong Y., Dijkwel P.P., Mueller-Roeber B., Gechev T.S. (2019). Genome-Wide Analysis of ROS Antioxidant Genes in Resurrection Species Suggest an Involvement of Distinct ROS Detoxification Systems during Desiccation. Int. J. Mol. Sci..

[58] Gao F., Chen J., Ma T., Li H., Wang N., Li Z., Zhang Z., Zhou Y. (2014). The Glutathione Peroxidase Gene Family in *Thellungiella*
*Salsuginea*: Genome-Wide Identification, Classification, and Gene and Protein Expression Analysis under Stress Conditions. Int. J. Mol. Sci..

[59] San Koh C., Didierjean C., Navrot N., Panjikar S., Mulliert G., Rouhier N., Jacquot J.-P., Aubry A., Shawkataly O., Corbier C. (2007). Crystal Structures of a Poplar Thioredoxin Peroxidase That Exhibits the Structure of Glutathione Peroxidases: Insights into Redox-Driven Conformational Changes. J. Mol. Biol..

[60] Toppo S., Vanin S., Bosello V., Tosatto S.C.E. (2008). Evolutionary and Structural Insights into the Multifaceted Glutathione Peroxidase (Gpx) Superfamily. Antioxid. Redox Signal..

[61] Herbette S., Lenne C., Leblanc N., Julien J., Drevet J.R., Roeckel-Drevet P. (2002). Two GPX-like Proteins from *Lycopersicon Esculentum* and *Helianthus Annuus* Are Antioxidant Enzymes with Phospholipid Hydroperoxide Glutathione Peroxidase and Thioredoxin Peroxidase Activities. Eur. J. Biochem..

[62] Jung B.G., Lee K.O., Lee S.S., Chi Y.H., Jang H.H., Kang S.S., Lee K., Lim D., Yoon S.C., Yun D.-J. (2002). A Chinese Cabbage CDNA with High Sequence Identity to Phospholipid Hydroperoxide Glutathione Peroxidases Encodes a Novel Isoform of Thioredoxin-Dependent Peroxidase. J. Biol. Chem..

[63] Seiler A., Schneider M., Förster H., Roth S., Wirth E.K., Culmsee C., Plesnila N., Kremmer E., Rådmark O., Wurst W. (2008). Glutathione Peroxidase 4 Senses and Translates Oxidative Stress into 12/15-Lipoxygenase Dependent-and AIF-Mediated Cell Death. Cell Metab..

[64] Bela K., Riyazuddin R., Horváth E., Hurton Á., Gallé Á., Takács Z., Zsigmond L., Szabados L., Tari I., Csiszár J. (2018). Comprehensive Analysis of Antioxidant Mechanisms in *Arabidopsis* Glutathione Peroxidase-like Mutants under Salt-and Osmotic Stress Reveals Organ-Specific Significance of the AtGPXL’s Activities. Environ. Exp. Bot..

[65] Chang C.C.C., Slesak I., Jordá L., Sotnikov A., Melzer M., Miszalski Z., Mullineaux P.M., Parker J.E., Karpinska B., Karpinski S. (2009). Arabidopsis Chloroplastic Glutathione Peroxidases Play a Role in Cross Talk between Photooxidative Stress and Immune Responses. Plant Physiol..

[66] Gaber A. (2011). *Arabidopsis* Glutathione Peroxidase 8 Is a Key Enzyme in Response to Environmental Stresses. Arab. J. Biotechnol..

[67] Riyazuddin R., Bela K., Horváth E., Rigó G., Gallé Á., Szabados L., Fehér A., Csiszár J. (2019). Overexpression of the Arabidopsis Glutathione Peroxidase-like 5 Gene (AtGPXL5) Resulted in Altered Plant Development and Redox Status. Environ. Exp. Bot..

[68] Toppo S., Flohé L., Ursini F., Vanin S., Maiorino M. (2009). Catalytic Mechanisms and Specificities of Glutathione Peroxidases: Variations of a Basic Scheme. Biochim. Biophys. Acta BBA-Gen. Subj..

[69] Riyazuddin R., Bela K., Poór P., Szepesi Á., Horváth E., Rigó G., Szabados L., Fehér A., Csiszár J. (2022). Crosstalk between the Arabidopsis Glutathione Peroxidase-Like 5 Isoenzyme (AtGPXL5) and Ethylene. Int. J. Mol. Sci..

[70] Dixon D.P., Edwards R. (2010). Glutathione Transferases. The Arabidopsis Book.

[71] Luo D., Smith S.W., Anderson B.D. (2005). Kinetics and Mechanism of the Reaction of Cysteine and Hydrogen Peroxide in Aqueous Solution. J. Pharm. Sci..

[72] Marinho H.S., Real C., Cyrne L., Soares H., Antunes F. (2014). Hydrogen Peroxide Sensing, Signaling and Regulation of Transcription Factors. Redox Biol..

[73] Diao Y., Xu H., Li G., Yu A., Yu X., Hu W., Zheng X., Li S., Wang Y., Hu Z. (2014). Cloning a Glutathione Peroxidase Gene from *Nelumbo*
*Nucifera* and Enhanced Salt Tolerance by Overexpressing in Rice. Mol. Biol. Rep..

[74] Passaia G., Fonini L.S., Caverzan A., Jardim-Messeder D., Christoff A.P., Gaeta M.L., de Araujo Mariath J.E., Margis R., Margis-Pinheiro M. (2013). The Mitochondrial Glutathione Peroxidase GPX3 Is Essential for H_2_O_2_ Homeostasis and Root and Shoot Development in Rice. Plant Sci..

[75] Delaunay A., Pflieger D., Barrault M.-B., Vinh J., Toledano M.B. (2002). A Thiol Peroxidase Is an H_2_O_2_ Receptor and Redox-Transducer in Gene Activation. Cell.

[76] Paiva A.L.S., Passaia G., Jardim-Messeder D., Nogueira F.C.S., Domont G.B., Margis-Pinheiro M. (2021). The Mitochondrial Isoform Glutathione Peroxidase 3 (OsGPX3) Is Involved in ABA Responses in Rice Plants. J. Proteom..

[77] Lima-Melo Y., Carvalho F.E.L., Martins M.O., Passaia G., Sousa R.H.V., Neto M.C.L., Margis-Pinheiro M., Silveira J.A.G. (2016). Mitochondrial GPX1 Silencing Triggers Differential Photosynthesis Impairment in Response to Salinity in Rice Plants. J. Integr. Plant Biol..

[78] Paiva A.L.S., Passaia G., Lobo A.K.M., Jardim-Messeder D., Silveira J.A.G., Margis-Pinheiro M. (2019). Mitochondrial Glutathione Peroxidase (OsGPX3) Has a Crucial Role in Rice Protection against Salt Stress. Environ. Exp. Bot..

[79] Li K., Jia Q., Guo J., Zhu Z., Shao M., Wang J., Li W., Dai J., Guo M., Li R. (2022). The High Chlorophyll Fluorescence 244 (HCF244) Is Potentially Involved in Glutathione Peroxidase 7-Regulated High Light Stress in *Arabidopsis*
*thaliana*. Environ. Exp. Bot..

[80] Gaber A., Ogata T., Maruta T., Yoshimura K., Tamoi M., Shigeoka S. (2012). The Involvement of *Arabidopsis* Glutathione Peroxidase 8 in the Suppression of Oxidative Damage in the Nucleus and Cytosol. Plant Cell Physiol..

[81] Luo C., Cai X.-T., Du J., Zhao T.-L., Wang P.-F., Zhao P.-X., Liu R., Xie Q., Cao X.-F., Xiang C.-B. (2016). PARAQUAT TOLERANCE3 Is an E3 Ligase That Switches off Activated Oxidative Response by Targeting Histone-Modifying PROTEIN METHYLTRANSFERASE4b. PLoS Genet..

[82] Yang X., Lin P., Luo Y., Bai H., Liao X., Li X., Tian Y., Jiang B., Pan Y., Zhang F. (2022). Lysine Decrotonylation of Glutathione Peroxidase at Lysine 220 Site Increases Glutathione Peroxidase Activity to Resist Cold Stress in *Chrysanthemum*. Ecotoxicol. Environ. Saf..

[83] Ganie S.A., Reddy A.S.N. (2021). Stress-Induced Changes in Alternative Splicing Landscape in Rice: Functional Significance of Splice Isoforms in Stress Tolerance. Biology.

[84] Zhai C.-Z., Zhao L., Yin L.-J., Chen M., Wang Q.-Y., Li L.-C., Xu Z.-S., Ma Y.-Z. (2013). Two Wheat Glutathione Peroxidase Genes Whose Products Are Located in Chloroplasts Improve Salt and H_2_O_2_ Tolerances in Arabidopsis. PLoS ONE.

[85] Alves A.M.M., Reis S.P.M., Gramacho K.P., Micheli F. (2020). The Glutathione Peroxidase Family of Theobroma Cacao: Involvement in the Oxidative Stress during Witches’ Broom Disease. Int. J. Biol. Macromol..

[86] Wang Y., Cao S., Sui X., Wang J., Geng Y., Gao F., Zhou Y. (2022). Genome-Wide Characterization, Evolution, and Expression Analysis of the Ascorbate Peroxidase and Glutathione Peroxidase Gene Families in Response to Cold and Osmotic Stress in *Ammopiptanthus*
*nanus*. J. Plant Growth Regul..

[87] Passaia G., Caverzan A., Fonini L.S., Carvalho F.E.L., Silveira J.A.G., Margis-Pinheiro M. (2014). Chloroplastic and Mitochondrial GPX Genes Play a Critical Role in Rice Development. Biol. Plant..

[88] Wu F., Jiang G., Yan H., Xiao L., Liang H., Zhang D., Jiang Y., Duan X. (2021). Redox Regulation of Glutathione Peroxidase by Thioredoxin in Longan Fruit in Relation to Senescence and Quality Deterioration. Food Chem..

[89] Bashandy T., Guilleminot J., Vernoux T., Caparros-Ruiz D., Ljung K., Meyer Y., Reichheld J.-P. (2010). Interplay between the NADP-Linked Thioredoxin and Glutathione Systems in *Arabidopsis* Auxin Signaling. Plant Cell.

[90] Diaz Vivancos P., Wolff T., Markovic J., Pallardo F.V., Foyer C.H. (2010). A Nuclear Glutathione Cycle within the Cell Cycle. Biochem. J..

[91] Aquilano K., Baldelli S., Ciriolo M.R. (2014). Glutathione: New Roles in Redox Signaling for an Old Antioxidant. Front. Pharmacol..

[92] Reichheld J.-P., Khafif M., Riondet C., Droux M., Bonnard G., Meyer Y. (2007). Inactivation of Thioredoxin Reductases Reveals a Complex Interplay between Thioredoxin and Glutathione Pathways in Arabidopsis Development. Plant Cell.

[93] Pagnussat G.C., Yu H.-J., Ngo Q.A., Rajani S., Mayalagu S., Johnson C.S., Capron A., Xie L.-F., Ye D., Sundaresan V. (2005). Genetic and Molecular Identification of Genes Required for Female Gametophyte Development and Function in Arabidopsis. Development.

[94] Marty L., Siala W., Schwarzländer M., Fricker M.D., Wirtz M., Sweetlove L.J., Meyer Y., Meyer A.J., Reichheld J.-P., Hell R. (2009). The NADPH-Dependent Thioredoxin System Constitutes a Functional Backup for Cytosolic Glutathione Reductase in Arabidopsis. Proc. Natl. Acad. Sci. USA.

[95] Lu J., Holmgren A. (2014). The Thioredoxin Antioxidant System. Free Radic. Biol. Med..

[96] Bela K., Bangash S.A.K., Csiszár J. (2017). Plant Glutathione Peroxidases: Antioxidant Enzymes in Plant Stress Responses and Tolerance. Glutathione in Plant Growth, Development, and Stress Tolerance.

[97] Jiang K., Moe-Lange J., Hennet L., Feldman L.J. (2016). Salt Stress Affects the Redox Status of *Arabidopsis* Root Meristems. Front. Plant Sci..

[98] Cheng J.-C., Seeley K.A., Sung Z.R. (1995). RML1 and RML2, *Arabidopsis* Genes Required for Cell Proliferation at the Root Tip. Plant Physiol..

[99] Vernoux T., Wilson R.C., Seeley K.A., Reichheld J.-P., Muroy S., Brown S., Maughan S.C., Cobbett C.S., Van Montagu M., Inzé D. (2000). The ROOT MERISTEMLESS1/CADMIUM SENSITIVE2 Gene Defines a Glutathione-Dependent Pathway Involved in Initiation and Maintenance of Cell Division during Postembryonic Root Development. Plant Cell.

[100] Frendo P., Harrison J., Norman C., Jiménez M.J.H., Van de Sype G., Gilabert A., Puppo A. (2005). Glutathione and Homoglutathione Play a Critical Role in the Nodulation Process of Medicago Truncatula. Mol. Plant-Microbe Interact..

[101] Schnaubelt D., Queval G., Dong Y., Diaz-Vivancos P., Makgopa M.E., Howell G., De Simone A., Bai J., Hannah M.A., Foyer C.H. (2015). Low Glutathione Regulates Gene Expression and the Redox Potentials of the Nucleus and Cytosol in *Arabidopsis thaliana*. Plant. Cell Environ..

[102] Sato A., Yamamoto K.T. (2008). Overexpression of the Non-canonical Aux/IAA Genes Causes Auxin-related Aberrant Phenotypes in *Arabidopsis*. Physiol. Plant..

[103] Mravec J., Skůpa P., Bailly A., Hoyerová K., Křeček P., Bielach A., Petrášek J., Zhang J., Gaykova V., Stierhof Y.-D. (2009). Subcellular Homeostasis of Phytohormone Auxin Is Mediated by the ER-Localized PIN5 Transporter. Nature.

[104] Hamaguchi A., Yamashino T., Koizumi N., Kiba T., Kojima M., Sakakibara H., Mizuno T. (2008). A Small Subfamily of Arabidopsis RADIALIS-LIKE SANT/MYB Genes: A Link to HOOKLESS1-Mediated Signal Transduction during Early Morphogenesis. Biosci. Biotechnol. Biochem..

[105] Meyer Y., Belin C., Delorme-Hinoux V., Reichheld J.-P., Riondet C. (2012). Thioredoxin and Glutaredoxin Systems in Plants: Molecular Mechanisms, Crosstalks, and Functional Significance. Antioxid. Redox Signal..

[106] Shahpiri A., Svensson B., Finnie C. (2008). The NADPH-Dependent Thioredoxin Reductase/Thioredoxin System in Germinating Barley Seeds: Gene Expression, Protein Profiles, and Interactions between Isoforms of Thioredoxin *h* and Thioredoxin Reductase. Plant Physiol..

[107] Benitez-Alfonso Y., Cilia M., Roman A.S., Thomas C., Maule A., Hearn S., Jackson D. (2009). Control of Arabidopsis Meristem Development by Thioredoxin-Dependent Regulation of Intercellular Transport. Proc. Natl. Acad. Sci. USA.

[108] Meng L., Wong J.H., Feldman L.J., Lemaux P.G., Buchanan B.B. (2010). A Membrane-Associated Thioredoxin Required for Plant Growth Moves from Cell to Cell, Suggestive of a Role in Intercellular Communication. Proc. Natl. Acad. Sci. USA.

[109] Arsova B., Hoja U., Wimmelbacher M., Greiner E., Üstün Ş., Melzer M., Petersen K., Lein W., Börnke F. (2010). Plastidial Thioredoxin z Interacts with Two Fructokinase-like Proteins in a Thiol-Dependent Manner: Evidence for an Essential Role in Chloroplast Development in *Arabidopsis* and *Nicotiana*
*benthamiana*. Plant Cell.

[110] Wang X., Fang G., Yang J., Li Y. (2017). A Thioredoxin-Dependent Glutathione Peroxidase (OsGPX5) Is Required for Rice Normal Development and Salt Stress Tolerance. Plant Mol. Biol. Report..

[111] Zhou B., Yao W., Wang S., Wang X., Jiang T. (2014). The Metallothionein Gene, TaMT3, from Tamarix Androssowii Confers Cd^2+^ Tolerance in Tobacco. Int. J. Mol. Sci..

[112] Sarıkaya E., Doğan S. (2020). Glutathione Peroxidase in Health and Diseases. Glutathione System and Oxidative Stress in Health and Disease.

[113] Chen Z., Hu T., Zhu S., Mukaisho K., El-Rifai W., Peng D.-F. (2017). Glutathione Peroxidase 7 Suppresses Cancer Cell Growth and Is Hypermethylated in Gastric Cancer. Oncotarget.

[114] Gaber A. (2014). The Importance of Arabidopsis Glutathione Peroxidase 8 for Protecting *Arabidopsis* Plant and *E. Coli* Cells against Oxidative Stress. GM Crops Food.

[115] Sugimoto M., Sakamoto W. (1997). Putative Phospholipid Hydroperoxide Glutathione Peroxidase Gene from *Arabidopsis thaliana* Induced by Oxidative Stress. Genes Genet. Syst..

[116] Herbette S., Le Menn A., Rousselle P., Ameglio T., Faltin Z., Branlard G., Eshdat Y., Julien J.-L., Drevet J.R., Roeckel-Drevet P. (2005). Modification of Photosynthetic Regulation in Tomato Overexpressing Glutathione Peroxidase. Biochim. Biophys. Acta BBA-Gen. Subj..

[117] Faltin Z., Holland D., Velcheva M., Tsapovetsky M., Roeckel-Drevet P., Handa A.K., Abu-Abied M., Friedman-Einat M., Eshdat Y., Perl A. (2010). Glutathione Peroxidase Regulation of Reactive Oxygen Species Level Is Crucial for in Vitro Plant Differentiation. Plant Cell Physiol..

